# Supplementary material for: Expanding the Applicability of an Innovative Laccase TTI in Intelligent Packaging by Adding an Enzyme Inhibitor to Change Its Coloration Kinetics
Source: Polymers (Basel). 2021 Oct 22;13(21):3646. doi: 10.3390/polym13213646 (PMC8587941; doi:10.3390/polym13213646)
Supplement: Supplementary file 1 [file polymers-13-03646-s001.zip › polymers-1404880-supplementary.pdf]

Supplementary Materials

1. The chemical structure of laccase and guaiacol.

(A)

(B)

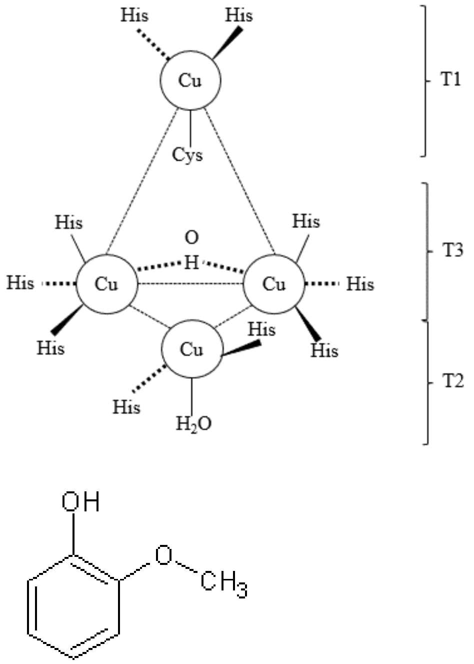

Supplementary Figure S1 The chemical structure of (A) laccase and (B) guaiacol.

2. Coloration lag was observed for NaN_3 added laccase TTI at the simulated cold chain temperature of 5 °C (Supplementary Fig. 2A). In other words, no colors were initially observed and the color that appeared was markedly deepened prior to approaching the coloration endpoint. When more NaN_3 was added or less laccase was immobilized, the coloration lag phase was extended. This finding indicated that when the ratio of enzyme inhibitor to immobilized enzyme was higher, the effect of hysteretic coloration was more pronounced and the time to reach coloration endpoint in the laccase TTI prototype was longer. Coloration lag of laccase TTI is less obvious at higher temperature environment (Supplementary Fig. 2B−D), indicating that NaN_3 inhibited laccase activity more significantly at low temperatures. Nevertheless, when the ambient temperature rises and deviates from the cold chain temperature, the TTI coloration can still be accelerated. This phenomenon indicated that the TTI is useful for managing food quality in the cold chain.

3.

(A)

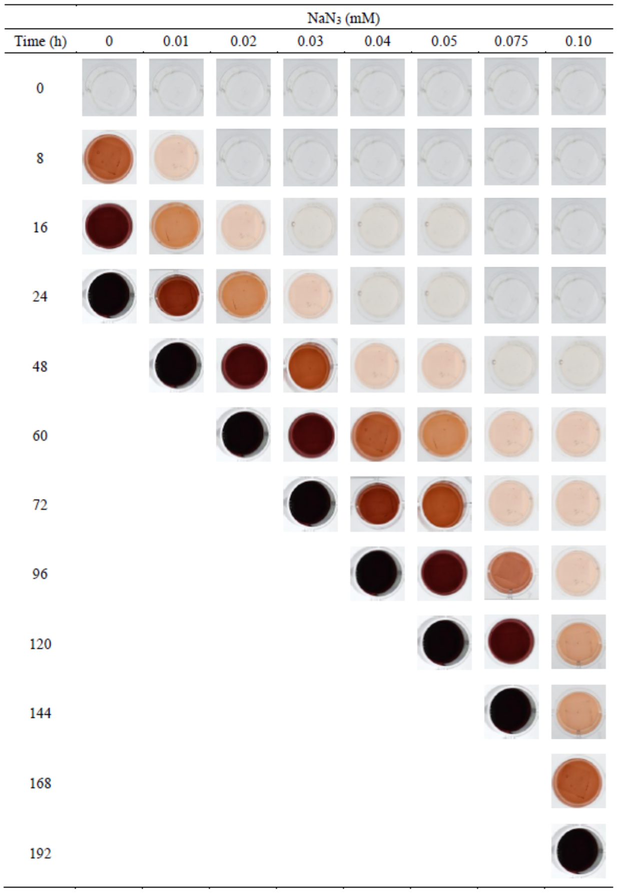
NaN3 (mM)
Time (h)
0
0.01
0.02
0.03
0.04
0.05
0.075
0.10
0
8
16
24
48
60
72
96
120
144
168
192

(B)

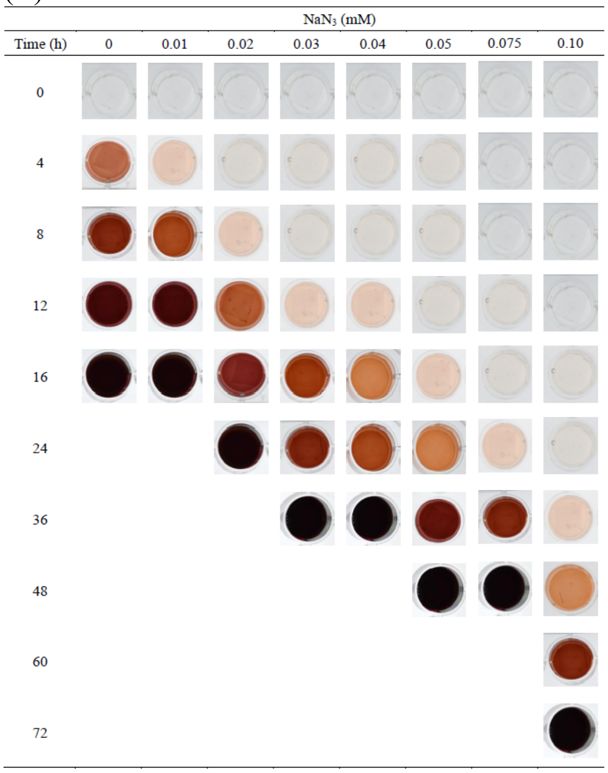
NaN3 (mM)
Time (h)
0
0.01
0.02
0.03
0.04
0.05
0.075
0.10
0
4
8
12
16
24
36
48
60
72

(C)

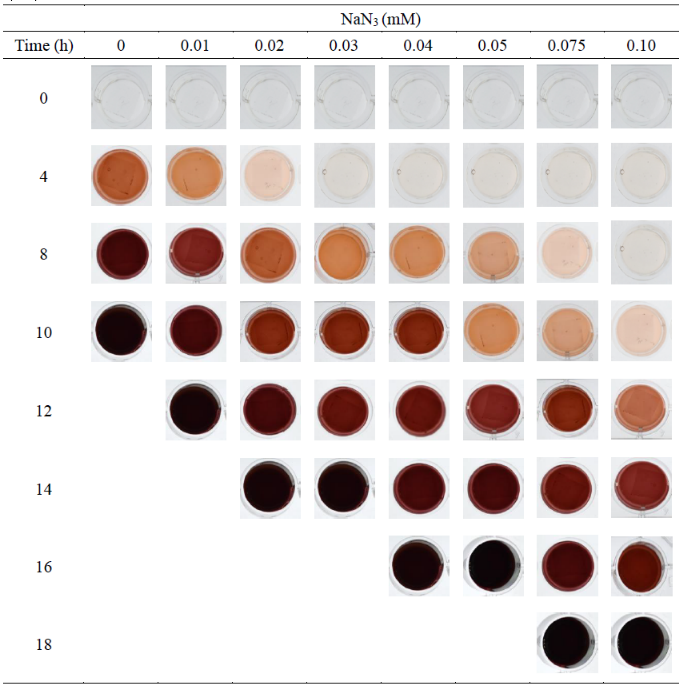

(D)

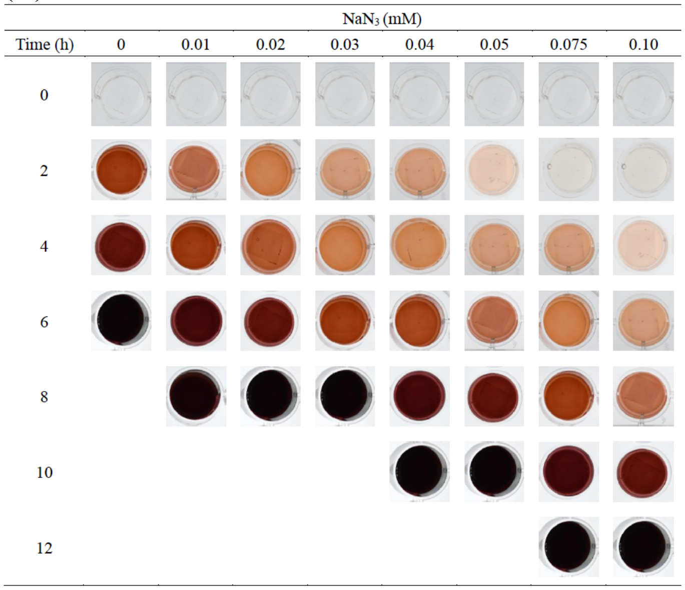

Supplementary Figure S2 Coloration shown by laccase TTI (20 μg/cm^2) prototypes during storage at (A) 5 °C, (B) 15 °C, (C) 25 °C, and (D) 35 °C.

4.

Supplementary Table S1. Coloration endpoint of laccase TTI prototype (with 15–25 μg/cm^2 laccase and 0–0.10 mM N-HGG).

A–C: Significant difference in coloration endpoint in the amount of enzyme immobilized; a–d: significant difference in coloration endpoint between environmental temperatures ($P < 0.05$).

**N-HGG (mM)**	***T* (°C)**	**Coloration endpoint (h)**		
		15 μg/cm^2 laccase	20 μg/cm^2 laccase	25 μg/cm^2 laccase
0	5	21.8 ± 0.6 ^{Aa}	14.1 ± 0.4 ^{Ba}	11.0 ± 0.8 ^{Ca}
	15	10.0 ± 0.4 ^{Ab}	9.5 ± 0.3 ^{Bb}	7.4 ± 0.3 ^{Cb}
	25	7.0 ± 0.1 ^{Ac}	4.9 ± 0.2 ^{Bc}	4.1 ± 0.1 ^{Cc}
	35	6.1 ± 0.3 ^{Ad}	4.0 ± 0.3 ^{Bd}	3.8 ± 0.4 ^{Bc}
0.01	5	31.2 ± 0.5 ^{Aa}	29.3 ± 2.8 ^{ABa}	19.2 ± 1.4 ^{Ca}
	15	12.9 ± 1.9 ^{Ab}	12.3 ± 0.5 ^{ABb}	9.9 ± 0.5 ^{Cb}
	25	7.7 ± 0.4 ^{Ac}	6.7 ± 0.1 ^{Bc}	6.2 ± 0.1 ^{Cc}
	35	7.0 ± 0.3 ^{Ad}	5.3 ± 0.1 ^{Bd}	5.0 ± 0.3 ^{Bd}
0.02	5	46.4 ± 1.4 ^{Aa}	41.0 ± 1.7 ^{Ba}	35.7 ± 0.5 ^{Ca}
	15	21.4 ± 0.8 ^{Ab}	15.3 ± 0.4 ^{Bb}	13.2 ± 0.6 ^{Cb}
	25	10.8 ± 0.1 ^{Ac}	9.8 ± 0.3 ^{Bc}	9.0 ± 0.4 ^{Cc}
	35	7.2 ± 0.6 ^{Ad}	6.2 ± 0.4 ^{Bd}	6.1 ± 0.1 ^{Bd}
0.03	5	79.5 ± 2.9 ^{Aa}	45.5 ± 0.9 ^{Ba}	45.3 ± 1.2 ^{Ba}
	15	30.8 ± 1.6 ^{Ab}	29.3 ± 3.8 ^{Ab}	14.6 ± 1.0 ^{Bb}
	25	14.8 ± 0.3 ^{Ac}	11.4 ± 0.9 ^{Bc}	11.2 ± 0.7 ^{Bc}
	35	7.5 ± 0.1 ^{Ad}	6.4 ± 0.5 ^{Bd}	6.3 ± 0.4 ^{Bd}
0.04	5	87.5 ± 2.6 ^{Aa}	78.6 ± 3.3 ^{Ba}	65.4 ± 1.6 ^{Ca}
	15	31.2 ± 1.0 ^{Ab}	30.1 ± 0.4 ^{ABb}	15.4 ± 0.2 ^{Cb}
	25	16.3 ± 1.8 ^{Ac}	15.8 ± 0.6 ^{Ac}	12.2 ± 0.8 ^{Bc}
	35	8.2 ± 0.1 ^{Ad}	6.9 ± 0.6 ^{Bd}	6.8 ± 0.5 ^{Bd}
0.05	5	90.2 ± 1.4 ^{Aa}	86.0 ± 0.5 ^{Ba}	69.8 ± 0.8 ^{Ca}
	15	32.6 ± 0.7 ^{Ab}	31.0 ± 0.4 ^{Bb}	16.1 ± 0.8 ^{Cb}
	25	17.7 ± 0.2 ^{Ac}	16.8 ± 0.3 ^{Bc}	13.2 ± 1.5 ^{Cc}
	35	9.0 ± 0.5 ^{Ad}	7.4 ± 0.0 ^{Bd}	7.2 ± 0.3 ^{Cd}
0.075	5	185.8 ± 1.4 ^{Aa}	168.0 ± 1.3 ^{Ba}	116.6 ± 0.4 ^{Ca}
	15	68.5 ± 0.2 ^{Ab}	45.2 ± 2.5 ^{Bb}	31.2 ± 1.2 ^{Cb}
	25	19.0 ± 1.5 ^{Ac}	17.1 ± 0.3 ^{Bc}	14.5 ± 0.7 ^{Cc}
	35	16.7 ± 0.1 ^{Ad}	11.5 ± 0.3 ^{Bd}	10.1 ± 0.1 ^{Cd}
0.10	5	199.5 ± 2.1 ^{Aa}	189.8 ± 0.8 ^{Ba}	167.7 ± 0.2 ^{Ca}
	15	106.4 ± 1.2 ^{Ab}	65.0 ± 0.7 ^{Bb}	46.7 ± 1.4 ^{Cb}
	25	22.1 ± 0.5 ^{Ac}	17.5 ± 0.4 ^{Bc}	15.6 ± 0.9 ^{Cc}
	35	18.8 ± 2.3 ^{Ad}	11.9 ± 1.4 ^{Bd}	11.7 ± 0.8 ^{Bd}
